# Capsaicin Enhances Glutamatergic Synaptic Transmission to Neonatal Rat Hypoglossal Motor Neurons via a TRPV1-Independent Mechanism

**DOI:** 10.3389/fncel.2017.00383

**Published:** 2017-12-05

**Authors:** Prajwal P. Thakre, Mark C. Bellingham

**Affiliations:** Faculty of Medicine, School of Biomedical Sciences, The University of Queensland, Brisbane, QLD, Australia

**Keywords:** excitatory synaptic transmission, hypoglossal motor neurons, capsaicin, TRPV1, intrinsic excitability, inhibitory synaptic transmission

## Abstract

We investigated whether capsaicin modulated synaptic transmission to hypoglossal motor neurons (HMNs) by acting on transient receptor potential vanilloid type 1 (TRPV1) receptors. Using whole-cell patch clamp recording from neonatal rat HMNs, we found that capsaicin increased spontaneous excitatory post-synaptic current (sEPSC) frequency and amplitude. Interestingly, the only effect of capsaicin on spontaneous inhibitory post-synaptic currents (sIPSCs) was a significant decrease in sIPSC amplitude without altering frequency, indicating a post-synaptic mechanism of action. The frequency of miniature excitatory post-synaptic currents (mEPSCs), recorded in the presence of tetrodotoxin (TTX), was also increased by capsaicin, but capsaicin did not alter mEPSC amplitude, consistent with a pre-synaptic mechanism of action. A negative shift in membrane current (I_holding_) was elicited by capsaicin under both recording conditions. The effect of capsaicin on excitatory synaptic transmission remained unchanged in the presence of the TRPV1 antagonists, capsazepine or SB366791, suggesting that capsaicin acts to modulate EPSCs via a mechanism which does not require TRPV1 activation. Capsaicin, however, did not alter evoked excitatory post-synaptic currents (eEPSCs) or the paired-pulse ratio (PPR) of eEPSCs. Repetitive action potential (AP) firing in HMNs was also unaltered by capsaicin, indicating that capsaicin does not change HMN intrinsic excitability. We have demonstrated that capsaicin modulates glutamatergic excitatory, as well as glycinergic inhibitory, synaptic transmission in HMNs by differing pre- and post-synaptic mechanisms. These results expand our understanding regarding the extent to which capsaicin can modulate synaptic transmission to central neurons.

## Key points

- Hypoglossal motor neurons were patch-clamped to record excitatory and inhibitory synaptic currents in neonatal rat brain slices.
- Capsaicin caused an increase in the frequency of spontaneous glutamatergic synaptic currents and induced an inward current in hypoglossal motor neurons in the presence and absence of tetrodotoxin.
- Two structurally different TRPV1 antagonists did not block these effects of capsaicin.
- Capsaicin, however, did not change motor neuron firing rate or evoked excitatory synaptic current amplitude and paired pulse ratio, and decreased glycinergic spontaneous inhibitory current amplitude without altering frequency in hypoglossal motor neurons.

## Introduction

Hypoglossal motor neurons (HMNs) innervate tongue muscles and play a critical role in control of oral behaviors like respiration, licking, suckling, mastication and vocalization (Berger et al., 1995). The dense dendritic arborization of HMNs (Núñez-Abades et al., 1994; Kanjhan et al., 2015) integrates afferent inputs from many brain regions (Altschuler et al., 1994; Fukunishi et al., 1999; Tarras-Wahlberg and Rekling, 2009), as well as afferent inputs from central and peripheral chemoreceptors and mechanoreceptors (Bailey and Fregosi, 2006). Neurons in the intermediate reticular formation act as an important source of premotor input to HMNs, including glutamatergic synaptic drive from the respiratory central pattern generation in the ventrolateral medulla oblongata (Koizumi et al., 2008; Koshiya et al., 2014). HMNs also receive excitatory serotonergic input from caudal raphe nuclei (Manaker and Tischler, 1993) and noradrenergic inputs from locus coeruleus (McBride and Sutin, 1976). Many neuroactive chemicals, including adenosine (Bellingham and Berger, 1994; Funk et al., 1997), acetylcholine (Bellingham and Berger, 1996; Ireland et al., 2012), serotonin (Singer et al., 1996) and noradrenaline (Parkis et al., 1995), can modulate these excitatory inputs to HMNs.

**Figure 1:**
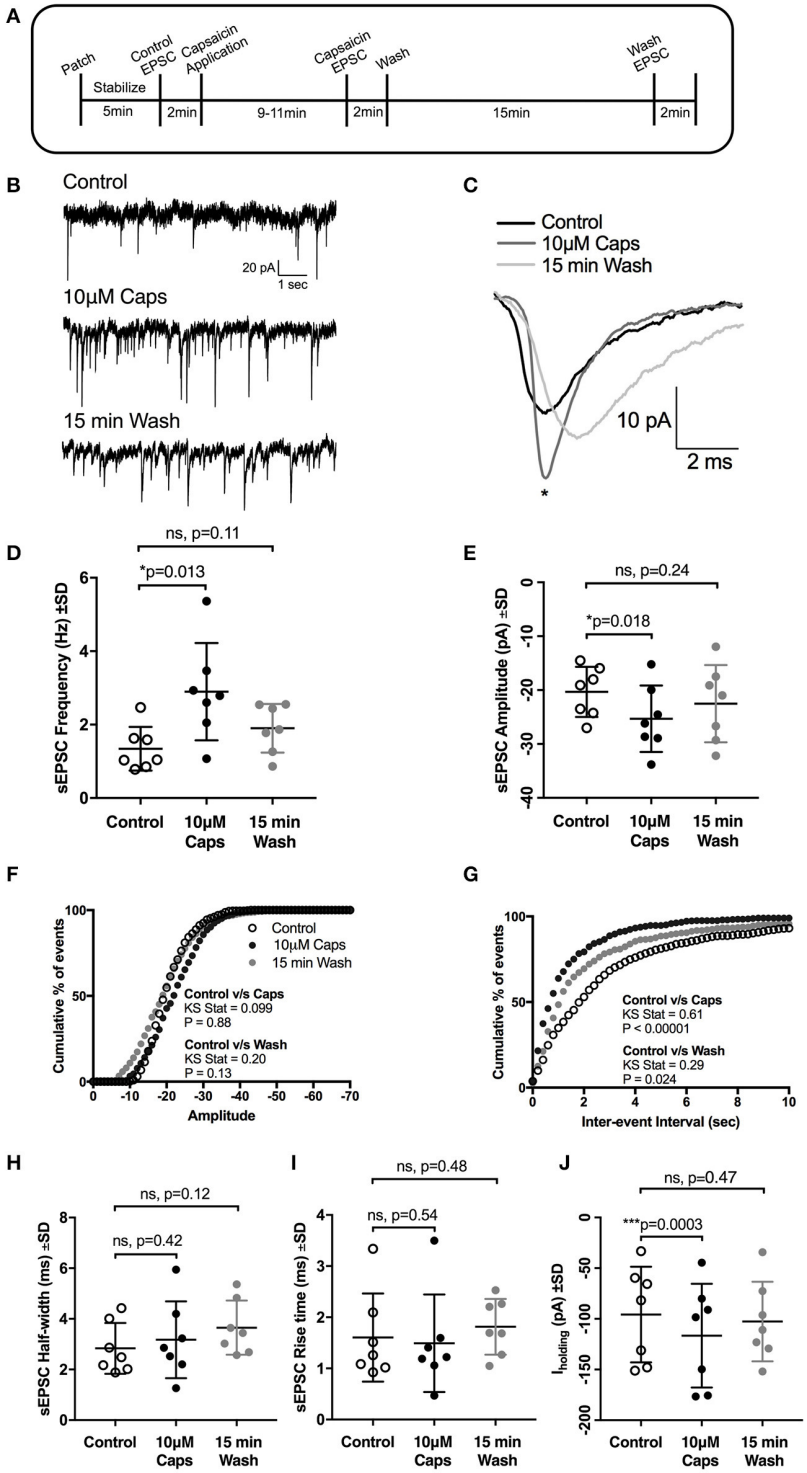
Capsaicin (Caps) increases glutamatergic synaptic transmission to rat HMNs. **(A)** A time line of the experimental protocol. After baseline control recording, slices were superfused with capsaicin (10 μM) dissolved in aCSF for 9–11 min before recording the effect of capsaicin (Caps) on spontaneous excitatory post-synaptic currents (sEPSCs), followed by a 15 min wash with aCSF devoid of Caps. **(B)** Representative sEPSCs recorded in the absence (control), and presence of 10 μM capsaicin, and after 15 min wash from a HMN voltage-clamped at −60 mV. **(C)** Representative averaged traces of sEPSCs before (control), during capsaicin and after wash. **(D)** Mean sEPSC frequency during control, capsaicin and wash conditions, showing a significant increase in sEPSC frequency with capsaicin. **(E)** Mean sEPSC amplitude during control, capsaicin and wash conditions, showing a significant increase in sEPSC amplitude with capsaicin. **(F,G)** Averaged cumulative frequency distribution plots of sEPSC amplitude **(F)** and inter-event interval **(G)** for HMNs in **(D,E)**. Kolmogorov-Smirnov test of inter-event interval shows a significant difference between control and capsaicin conditions. **(H,I)** Mean sEPSC half-width **(H)** and rise time **(I)** remain unchanged after capsaicin application. **(J)** Baseline holding current (I_holding_) was significantly altered in inward direction by capsaicin. All recordings were made in the presence of 20 μM strychnine HCl. Data represented as mean ± S.D. for *n* = 7 cells, each from a separate brain slice. Significance is shown as *p < 0.05, ***p < 0.001. Paired two-tailed *t*-test was used to determine statistical significance.

The coordination of HMN activity with other upper airway muscles during oral behaviors suggests that visceral and nociceptive afferent feedback are likely to modulate HMNs or their inputs (Fregosi and Ludlow, 2014). Pulmonary and airway vagal afferents terminate extensively on, and activate, neurons in the nucleus tractus solitarius (NTS) and area postrema (Beaumont et al., 2017); these central neurons then project directly or indirectly to HMNs (Travers and Norgren, 1983; Bailey and Fregosi, 2006) (Figure 1A). One distinguishing property of many vagal afferents is expression of the transient receptor potential vanilloid type 1 (TRPV1) receptor (Doyle et al., 2002; Hermes et al., 2016), a calcium permeable non-selective cation channel that is activated by the vanilloid capsaicin (Caterina et al., 1997; Messeguer et al., 2006), acidic pH (<6) (Tominaga et al., 1998), heat (Caterina et al., 1997) or membrane depolarization (Voets et al., 2004). In the periphery, capsaicin stimulates TRPV1 channels in a subset of sensory afferent neurons, causing excitation and local release of inflammatory mediators (Caterina et al., 1997, 2000). Activation of TRPV1 by capsaicin is known to initiate depolarization by the influx of sodium and calcium ions in peripheral sensory nerves, causing action potential generation and propagation to the brainstem and spinal cord, ultimately giving rise to burning or itching sensations (Anand and Bley, 2011).

The presence of TRPV1 on the central terminals of sensory afferents in the spinal cord and brainstem, as well as in several brain regions, including substantial nigra, locus coeruleus, hippocampus, nucleus tractus solitarius, periaqueductal gray and amygdala (Mezey et al., 2000; Roberts et al., 2004; Tóth et al., 2005; Sun et al., 2009; Aguiar et al., 2015) is consistent with the results of electrophysiological studies demonstrating TRPV1-mediated effects of capsaicin in these areas (Marinelli et al., 2002, 2003; Zschenderlein et al., 2011; Lu et al., 2017). These studies indicate that activation of TRPV1 by capsaicin can either modulate or directly release neurotransmitters in central neurons.

However, whether TRP channels can modulate these excitatory inputs to HMNs remains a relatively unexplored question. In anesthetized rats, the administration of capsaicin directly into the right atrium to excite vagal pulmonary C fibers, has shown to lower hypoglossal nerve activity at low doses, while higher doses reduce phasic hypoglossal activity but increase tonic hypoglossal discharge (Lee et al., 2003), while capsaicin administration in the jugular vein suppresses HMN activity (Lee et al., 2007). Moreover, capsaicin has also been reported to modulate synaptic transmission to an adjacent brainstem structure, the dorsal motor nucleus of the vagus (DMVs) (Xu and Smith, 2015) via a pre-synaptic mechanism. Most recently, capsaicin has been shown to modulate central respiratory rhythmic activity in medullary brain slices (Beaumont et al., 2017). However, the direct effects of capsaicin on synaptic inputs to HMNs have not been reported. We therefore investigated whether capsaicin can directly modulate synaptic transmission to HMNs.

## Materials and methods

### Brainstem slice preparation

All experimental procedures were performed after approval from The University of Queensland Animal Ethics Anatomical Biosciences Committee (approval SBMS/142/15/NHMRC), and were in accordance with the Australian Code for the Care and Use of Animals for Scientific Purposes, 8th Edition (National Health and Medical Research Council, 2013), as well as the Queensland Government Animal Research Act 2001, and associated Animal Care and Protection Regulations (2002 and 2008).

Wistar rats (7–14 day old, either sex) were used. Sodium pentobarbitone (60–80 mg/kg, i.p.) was administered intraperitoneally to anaesthetize the animals; the skull was rapidly removed and the brainstem dissected out into ice-cold high Mg^2+^ artificial cerebrospinal fluid (aCSF), which contained (in mM) 130 NaCl, 26 NaHCO_3_, 3 KCl, 5 MgCl_2_, 1 CaCl_2_, 1.25 NaH_2_PO_4_, 10 D-glucose (Bellingham and Berger, 1996; Bellingham, 2013). All solutions were bubbled with carbogen (95% O_2_ + 5% CO_2_) to maintain a pH of 7.4. The rostral brainstem end was fixed on a chuck using cyanoacrylate glue, with the ventral surface of the brainstem supported by an agar block glued to the chuck. The chuck with tissue was submerged in ice-cold high (5 mM) Mg^2+^ aCSF within a chamber, surrounded by an ice/water slurry. Transverse brainstem slices (300 μm thickness) were cut using a vibratome (Leica VT 1200 S, Leica Biosystems). Each rat yielded 3-4 slices. After cutting, slices were transferred to a holding chamber filled with the same high Mg^2+^ aCSF at 37°C, in a water bath for 45–60 min. Finally, slices were transferred to a new holding chamber containing recording aCSF (composition as above, except for 2 mM Ca^2+^, 1 mM Mg^2+^) and equilibrated for at least 30 min before recording at room temperature (21–22°C).

### Electrophysiology

Individual slices were placed in a recording chamber (volume 200 μL) and continuously superfused (1–2 ml/min) with recording aCSF throughout the experiment. HMNs were identified using a 60X water-immersion objective, an infrared video camera (Hamamatsu, Japan) and Nomarski optics on a Nikon E600FN microscope (Tokyo, Japan); all HMNs were ventral and lateral to the central canal, within the hypoglossal motor nucleus, and had a large soma. Whole cell patch clamp recordings were made using an Axopatch 1D amplifier (Axon Instruments, Foster City, CA, USA). Synaptic current recordings were sampled at 10 kHz, and low-pass filtered at 2 kHz, while a higher sampling rate (50 kHz) and low-pass filter (10 kHz) setting were used for action potential recordings; data was sampled and stored on a Windows XP computer, using PClamp 10.2 software and a Digidata 1332A digitizer (Axon Instruments). After advancing the electrode to the cell body, so that electrode solution ejected by positive pressure produced a small dimple, positive pressure was released and mild suction was applied until a stable membrane seal (resistance >2 GΩ) was achieved. Stronger suction was then applied until the cell membrane was broken. Cells were voltage-clamped at membrane potentials of −60 mV.

The patch pipette internal solution for recording excitatory and inhibitory post-synaptic currents (EPSCs/IPSCs) contained (in mM) 120 CsCl, 4 NaCl, 4 MgCl_2_, 0.001 CaCl_2_, 10 Cs N-2-hydroxyethyl-piperazine-N'-2-ethanesulfonic acid (HEPES), 10 caesium ethylene glycol-bis(β-aminoethyl ether)-N,N,N,N-tetra-acetic acid (EGTA), pH adjusted to 7.2 with CsOH. The internal solution for recording action potentials contained (in mM) 135 K methyl sulfate, 8 NaCl, 10 HEPES, and 0.3 EGTA; pH 7.2 with KOH. Pipette solution osmolarity was 290–300 mOsM, measured using a vapor pressure osmometer (Wescor). 3 adenosine 5′- triphosphate (ATP-Mg) and 0.3 guanosine 5-triphosphate-tris(hydroxymethyl) aminomethane (GTP-Tris) were added to the internal solution just before use. For recording glutamatergic EPSCs, strychnine HCl (20 μM) was added to all external recording solutions to block both glycine and GABA_A_ receptor-mediated inhibitory synaptic currents in HMNs. Similarly, for recording glycinergic IPSCs, NBQX (10 μM), APV (50 μM), and bicuculline (5 μM) were added to the recording buffer. To isolate miniature synaptic currents, tetrodotoxin (TTX) at a final concentration of 1 μM was added to the recording buffer. For evoked EPSC recordings, a bipolar concentric stimulation electrode (Frederick Haer) was placed in the reticular formation near the ventrolateral border of the hypoglossal nucleus, and a pair of stimulus currents of 0.5–1.1 mA and 0.1 ms duration was applied to evoke an EPSC with consistent first pulse EPSC amplitudes; inter-stimulus interval was 150 ms (Bellingham, 2013). Action potential (AP) firing was achieved by repetitive depolarizing current steps (2–120 pA) of 400 ms duration from a membrane potential of −65 mV in current clamp mode (Bellingham, 2013). Input resistance was calculated from the steady state amplitude of a brief (40 ms) negative (−10 pA) current step prior to the depolarizing step.

### Drug application

All chemicals were from Sigma Aldrich (St Louis, MO, USA) except tetrodotoxin (TTX, Alomone Labs, Israel). The final concentrations of drugs were prepared just before each experiment. Capsaicin was dissolved in ethanol, capsazepine in methanol and SB366791 in DMSO to make stock solutions, which were diluted using recording aCSF to final concentrations of 10 μM (capsaicin and capsazepine) and 20 μM (SB366791). We chose these concentrations to achieve maximal capsaicin responses (Gavva et al., 2004) and for comparison to other electrophysiological studies of capsaicin effects. The reservoir containing SB366791 was protected from light by aluminum foil. Final concentration of all solvents was ≤ 0.2%.

### Data analysis

Spontaneous and miniature synaptic currents were analyzed from one or more continuous data recordings of 2 min. Shape parameters (amplitude, 10–90% rise time, half-width) analysis was done in Clampfit 10.2 (Axon Instruments), using a template created by averaging visual detected individual synaptic events. Baseline holding current (I_holding_) and frequency of EPSCs for spontaneous recordings and input resistance for evoked recordings were also recorded. Paired-pulse ratio (PPR) was calculated as the mean peak amplitude of the averaged second evoked EPSC divided by the mean peak amplitude of the averaged first evoked EPSC. For action potential analysis, action potentials were detected and their frequency and shape parameters measured using Clampfit.

Results are expressed as mean±SD, unless otherwise stated, and changes were determined to be statistically significant at *P* < 0.05 by a paired two-tailed *t*-test, except where indicated, using Prism 7 (GraphPad). The cumulative frequency distributions of synaptic currents was compared using a two-sample Kolmogorov-Smirnov test.

## Results

### Capsaicin increases amplitude and frequency of spontaneous EPSCs

Earlier reports have suggested that capsaicin increases glutamatergic synaptic transmission in central neurons (Marinelli et al., 2002, 2003; Sikand and Premkumar, 2007). We therefore examined whether capsaicin altered spontaneous glutamatergic excitatory synaptic transmission (Figure 1A, sEPSCs) to HMNs. During capsaicin application (9–11 min, 10 μM), there was a significant increase in sEPSC frequency and amplitude (Figures 1B–G, Table 1). Increased sEPSC frequency is illustrated by the representative traces in Figure 1B. Figure 1C represents averaged spontaneous EPSCs from the three time periods shown, illustrating increased sEPSC amplitude with capsaicin and a partial washout of this effect. Mean sEPSC amplitude increased from −20.3 to −25.3 pA (+24% of control, *p* = 0.018, *n* = 7; Figure 1E). Mean sEPSC frequency was also increased from 1.34 to 2.89 Hz (+115% of control, *p* = 0.013; Figure 1D). A washout of 15 min partially restored sEPSC amplitude and frequency toward control values. The distribution of sEPSC amplitude showed a shift to higher amplitude events in the presence of capsaicin (Figure 1F). Similarly, the distribution of sEPSC inter-event intervals shows a significant shift toward smaller inter-event intervals (equating to higher frequency), which returned toward control values upon washout (Figure 1G). Other sEPSC shape parameters, such as half-width (Figure 1H) and 10–90% rise time (Figure 1I) remained unchanged during capsaicin application. Baseline holding current (I_holding_), however, did show a significant inward shift from −95.75 to −116.6 pA (+21% of control, *p* = 0.0003, Figure 1J, Table 1), and this effect also partially recovered after washout.

**Table 1:** Spontaneous EPSC parameters of HMNs upon application of capsaicin.

**Effect of Capsaicin on spontaneous EPSCs**											
**Parameter**	**Control**			**10 μM Caps**			**15 min Wash**			**Statistical significance Control vs. 10 μM Caps**	**Statistical significance Control vs. 15 min Wash**
	**Mean**	**±SD**	***n***	**Mean**	**±SD**	***n***	**Mean**	**±SD**	***n***		
Amplitude (pA)	−20.3	4.6	7	−25.3	6.1	7	−22.5	7.1	7	^*^p = 0.018	ns, *p* = 0.24
Half-width (ms)	2.8	1.0	7	3.1	1.5	7	3.6	1.0	7	ns, *p* = 0.42	ns, *p* = 0.12
Rise time (ms)	1.6	0.8	7	1.4	0.9	7	1.8	0.5	7	ns, *p* = 0.54	ns, *p* = 0.48
Frequency (Hz)	1.34	0.5	7	2.89	1.3	7	1.90	0.6	7	^*^p = 0.013	ns, *p* = 0.11
I_holding_ (pA)	−95.75	47.1	7	−116.6	51.1	7	−102.6	39.2	7	^***^p = 0.0003	ns, *p* = 0.47

### Effect of capsaicin on frequency of excitatory postsynaptic currents is mediated via a TTX-insensitive mechanism

**Figure 2:**
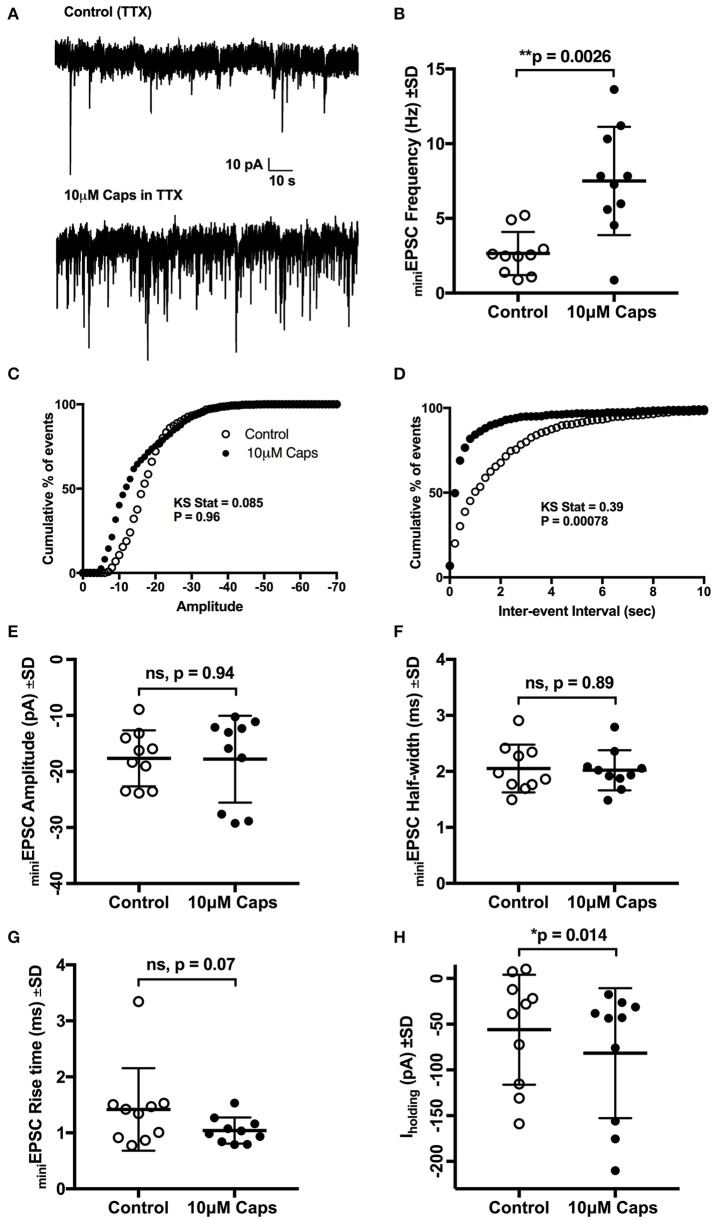
Capsaicin (Caps) induced increase in sEPSC frequency but not amplitude is mediated via non-TTX sensitive mechanism. All recordings were made in the presence of 1 μM tetrodotoxin (TTX) and 20 μM strychnine HCl. **(A)** Representative mEPSCs recorded in the absence (control) and presence of 10 μM capsaicin from a HMN voltage-clamped at −60 mV. **(B)** mEPSC frequency shows a significant increase during capsaicin. **(C,D)** Averaged cumulative frequency distribution plots of mEPSC amplitude **(C)** and inter-event interval **(D)** for the HMNs in **(B)**. Kolmogorov-Smirnov test applied to inter-event interval shows a significant difference between control and capsaicin. **(E–G)** shows amplitude half-width and rise time of mEPSCs which were unchanged during capsaicin application. **(H)** Baseline holding current (I_holding_) was shifted inwards with capsaicin. Data represented as mean ± S.D. for *n* = 10 cells, each from a separate brain slice. Significance is shown as ^*^p < 0.05, ^**^p < 0.01. Paired two-tailed *t*-test was used to determine statistical significance.

Capsaicin-induced changes in sEPSCs can either be an effect on action potential-independent spontaneous release of glutamate, or may, in part, be mediated via action potential-mediated release of neurotransmitters. Hence, to further test the effect of capsaicin on excitatory synaptic transmission in the absence of spontaneous action potential generation, we analyzed miniature EPSCs (mEPSCs) where, in addition to strychnine, 1 μM tetrodotoxin (TTX) was added to completely block action potentials (Figure 2A). Mean mEPSC frequency was still significantly increased by capsaicin, from 2.65 to 7.5 Hz (+183% from control, *p* = 0.0026, *n* = 10, Figure 2B, Table 2). However, there was no change in mEPSC amplitude after capsaicin application (Figure 2E). Figures 2C,D show the distribution of mEPSC amplitudes and inter-event intervals in control and capsaicin conditions. The amplitude distribution shows no significant change (Figure 2C), however, inter-event interval distribution shows a significant shift toward higher frequency (Figure 2D, Kolmogorov-Smirnov test). As for sEPSCs, there were no significant changes in mEPSC half-width (Figure 2F) and 10–90% rise time (Figure 2G). Baseline holding current (I_holding_) again showed a significant inward shift from −55.99 to −81.72 pA (+45% of control, *p* = 0.013, *n* = 10, Figure 2H, Table 2). Together, these effects of capsaicin on sEPSCs and mEPSCs show that capsaicin acts by increasing the release probability of pre-synaptic terminals or by modulating the excitability of presynaptic neurons making excitatory inputs to HMNs.

**Table 2:** Miniature EPSC parameters of HMNs upon application of capsaicin.

**Effect of Capsaicin on miniature EPSCs**							
**Parameter**	**Control**			**10 μM Caps**			**Statistical significance**
	**Mean**	**±SD**	***n***	**Mean**	**±SD**	***n***	
Amplitude (pA)	−17.6	4.9	10	−17.8	7.7	10	ns, *p* = 0.94
Half-width (ms)	2.0	0.4	10	2.0	0.3	10	ns, *p* = 0.89
Rise time (ms)	1.4	1.0	10	0.7	0.2	10	ns, *p* = 0.07
Frequency (Hz)	2.65	1.4	10	7.50	3.6	10	^**^p = 0.0026
I_holding_ (pA)	−55.99	60.1	10	−81.72	71.0	10	^*^p = 0.014

### TRPV1 antagonists do not block the effect of capsaicin on sEPSC frequency

**Figure 3:**
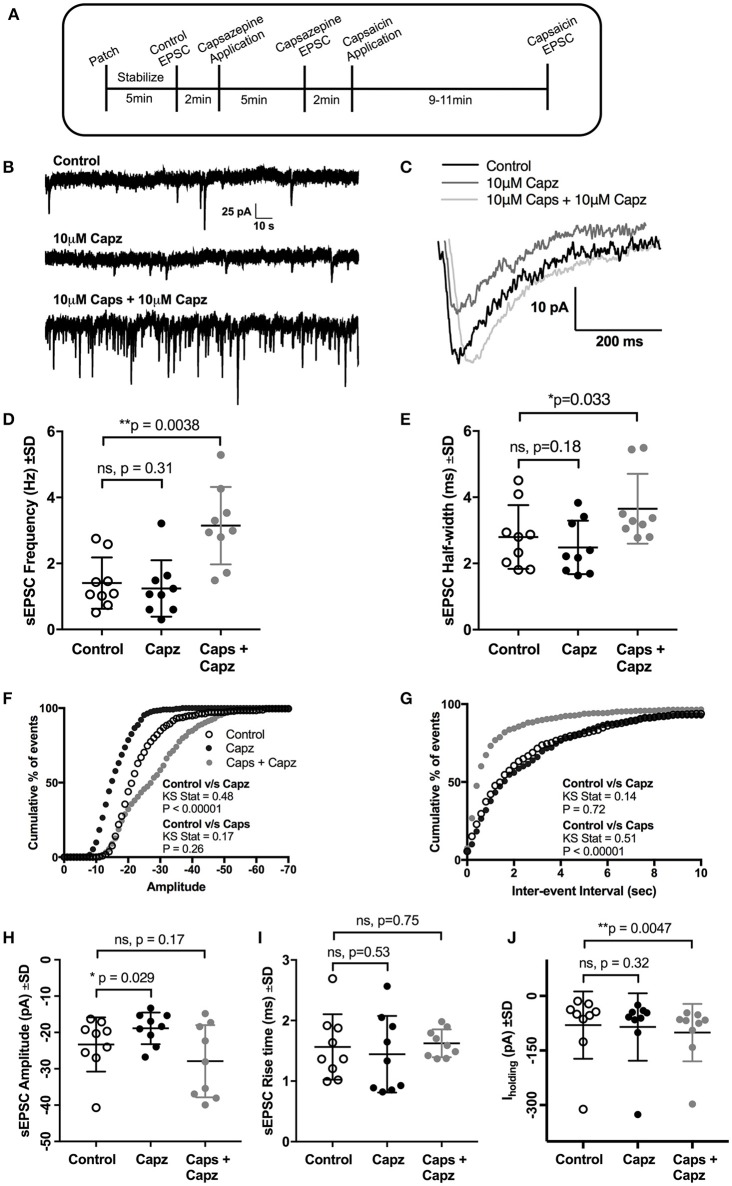
Pre-application of the TRPV1 antagonist, capsazepine (Capz), blocked the effect of capsaicin (Caps) on sEPSC amplitude but not on frequency. **(A)** Experimental protocol timing. After recording baseline control activity, slices were superfused with capsazepine (Capz, 10 μM) in aCSF for 5 min before recording capsazepine-induced changes in sEPSCs. Finally, capsaicin (10 μM) in aCSF was added for 9–11 min after which capsaicin induced sEPSC responses were recorded. Representative traces for sEPSC frequency **(B)** and amplitude **(C)** under control, capsazepine and capsaicin conditions. **(D)** Averaged cumulative frequency plots showing significant increase after capsaicin application even in presence of capsazepine. Mean sEPSC frequency **(D)** and half-width **(E)** showed significant increases after capsaicin application in presence of capsazepine. Averaged cumulative frequency distribution plots of sEPSC amplitude **(F)** and inter-event interval **(G)**. Kolmogorov-Smirnov test shows significant difference between control vs. capsazepine condition in amplitude plot and a significant difference between control and capsaicin condition in inter-event interval plot. **(H)** Mean sEPSC amplitude scatter plot shows a significant decrease in amplitude after capsazepine alone, but no change after capsaicin application. **(I)** Mean sEPSC rise time was not altered after capsazepine or capsaicin application. **(J)** Mean baseline I_holding_ was significantly altered after capsaicin, but not after capsazepine application alone. All recordings were made in the presence of 20 μM strychnine HCl. Data represented as mean ± S.D. for *n* = 9 cells, each from a separate brain slice. Significance is shown as ^*^p < 0.05, ^**^p < 0.01. Paired two-tailed *t*-test was used to determine statistical significance.

Next, we investigated if the actions of capsaicin are mediated via TRPV1 activation, by testing whether pre-application of the TRPV1 antagonist capsazepine (Urban and Dray, 1991) could block the effect of capsaicin on glutamatergic excitatory transmission to rat HMNs. Earlier reports have suggested that application of capsaicin in brain slice preparations (Marinelli et al., 2002) and cultured neurons (Sikand and Premkumar, 2007) increased excitatory transmission through activation of TRPV1 receptors and that this effect was abolished by capsazepine. Application of capsaicin in the presence of capsazepine (Figure 3A) still increased sEPSC frequency (+123% of control, *p* = 0.0038, *n* = 9; Figure 3D, Table 3) and I_holding_ (+25% of control, *p* = 0.0047, *n* = 9; Figure 3J, Table 3), but had no effect on amplitude (Figure 3H). Figure 3B represents the increase in frequency after addition of capsaicin in presence of capsazepine. Figure 3C shows averaged amplitude under 3 different conditions (control, capsazepine and capsazepine + capsaicin) respectively. It was interesting to note that capsaicin in presence of capsazepine increases the half-width of spontaneous currents (+30% of control, *p* = 0.032, *n* = 9; Figure 3E). The amplitude distribution of sEPSC showed a significant shift toward lower amplitude upon capsazepine application, but showed no further change after capsaicin application (Figure 3F). Also, the distribution of inter-event intervals showed no change between control and capsazepine, but showed a significant shift toward shorter inter-event intervals upon addition of capsaicin (Figure 3G). Capsazepine alone did not cause any significant effect on sEPSC frequency (Figure 3D), half-width (Figure 3E), rise time (Figure 3I) and I_holding_ (Figure 3J); however, there was a significant decrease in sEPSC amplitude (−19% of control, *p* = 0.028, *n* = 9, Figure 3H, Table 3), indicating a partial tonic effect of TRPV1 on sEPSC amplitude.

**Table 3:** Spontaneous EPSC parameters of HMNs upon application of capsaicin in presence of capsazepine.

**Pre-application of capsazepine (Capz) on effect of capsaicin (Caps)**											
**Parameter**	**Control**			**10 μM Capz**			**10 μM Caps +10 μM Capz**			**Statistical Significance Control vs. 10 μM Capz**	**Statistical Significance Control vs. 10 μM Caps**
	**Mean**	**±SD**	***n***	**Mean**	**±SD**	***n***	**Mean**	**±SD**	***n***		
Amplitude (pA)	−23.3	7.4	9	−18.8	4.3	9	−27.9	9.9	9	^*^p = 0.029	ns, *p* = 0.17
Half-width (ms)	2.8	0.9	9	2.4	0.8	9	3.6	1.0	9	ns, *p* = 0.18	^*^p = 0.033
Rise time (ms)	1.5	0.5	9	1.4	0.6	9	1.6	0.2	9	ns, *p* = 0.53	ns, *p* = 0.75
Frequency (Hz)	1.40	0.7	9	1.24	0.8	9	3.14	1.1	9	ns, *p* = 0.31	^**^p = 0.0038
I_holding_ (pA)	−80.26	92.5	9	−85.4	92.6	9	−100.7	78.9	9	ns, *p* = 0.32	^**^p = 0.0047

**Figure 4:**
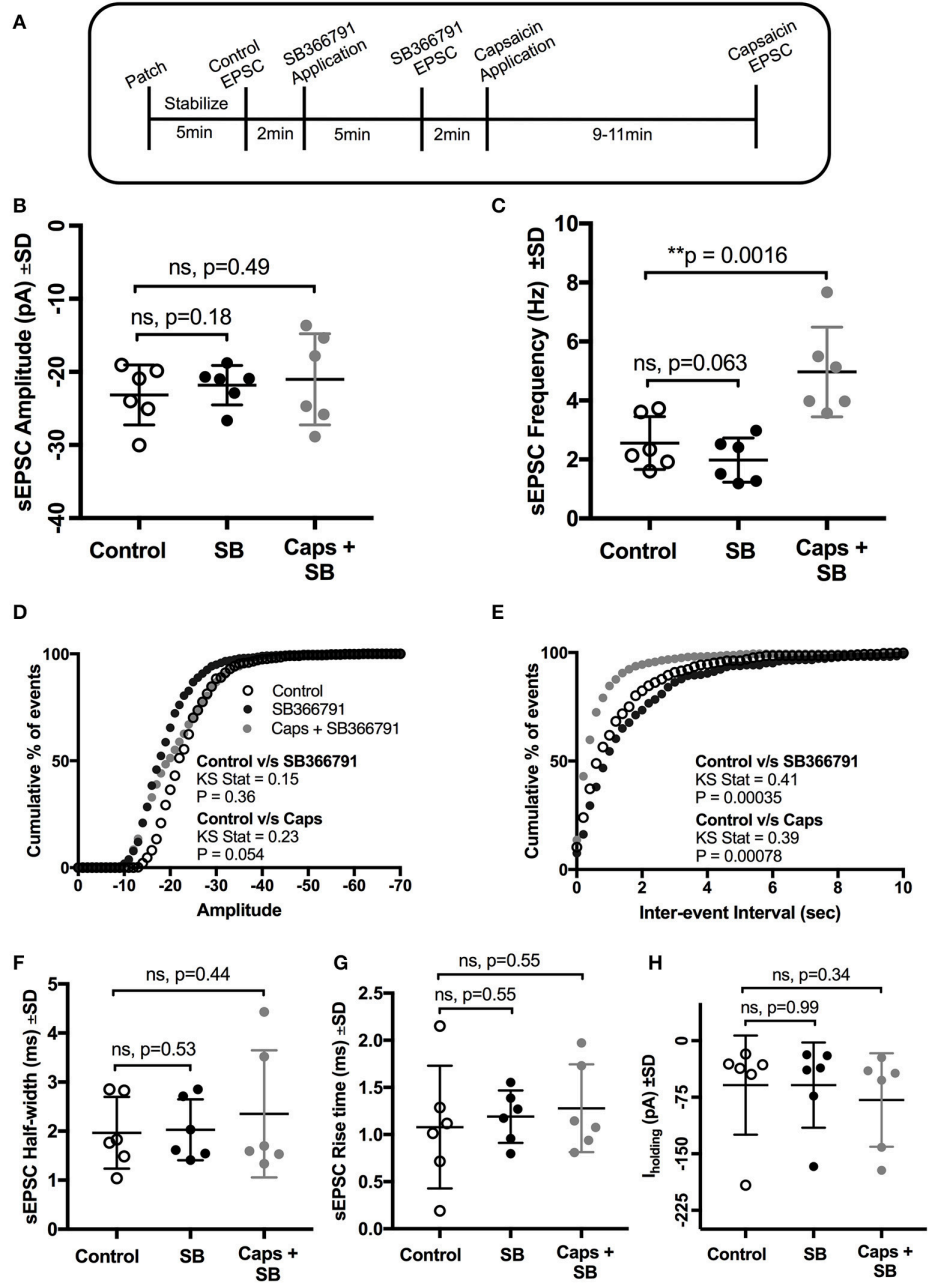
Pre-application of the TRPV1 antagonist, SB366791, blocked the effect of capsaicin (Caps) on sEPSC amplitude but not on frequency. **(A)** Experimental protocol timing. After recording baseline control activity, slices were superfused with SB366791 (20 μM) prepared in aCSF for 5 min before recording SB366791 induced sEPSC responses. Finally, capsaicin (10 μM) prepared in aCSF was added for 9–11 min, and then capsaicin induced sEPSC activity was recorded. **(B)** Mean amplitude scatter plot shows no difference between control, SB366791 or capsaicin conditions. **(C)** Mean frequency scatter plot shows a significant change after capsaicin, but no change with SB366791 application alone. Averaged cumulative frequency distribution plots of sEPSC amplitude **(D)** and inter-event interval **(E)**. Kolmogorov-Smirnov test of inter-event intervals shows a significant difference between control vs. SB366791 and control vs. capsaicin conditions. sEPSC half-width **(F)**, rise time **(G)** and baseline holding current **(H)** remained unchanged under SB366791 or capsaicin conditions. All recordings were made in the presence of 20 μM strychnine HCl. Data represented as mean ± S.D. for *n* = 6 cells, each from separate brain slice. Significance is shown as ^**^p < 0.01. Paired two-tailed *t*-test was used to determine statistical significance.

We therefore used a structurally different, selective, and potent TRPV1 antagonist, SB366791 (Gunthorpe et al., 2004; Varga et al., 2005), in order to further test the involvement of TRPV1 receptors in the actions of capsaicin. Earlier reports have shown that SB366791 antagonized capsaicin-induced changes in brain slice preparations (Gunthorpe et al., 2004; Varga et al., 2005). As for capsazepine, SB366791 (Figure 4A) did not alter the effect of capsaicin on sEPSC frequency (+94% of control, *n* = 6, *p* = 0.063; Figure 4C, Table 4), but did block the effect of capsaicin on sEPSC amplitude (Figure 4B) and I_holding_ (Figure 4H). The averaged sEPSC amplitude distribution showed no significant change between either control-SB366791 or control-capsaicin conditions (Figure 4D). Similarly, the averaged inter-event intervals distribution showed no change with SB366791, but showed a significant shift in events toward higher frequency when capsaicin was added (Figure 4E). No significant changes were observed in sEPSC half-width (Figure 4F) and 10–90% rise time (Figure 4G) under all conditions tested. By contrast to capsazepine, which decreased sEPSC amplitude, SB366791 by itself had no effect on sEPSC amplitude, indicating absence of any tonic SB366791-sensitive activity.

**Table 4:** Spontaneous EPSC parameters of HMNs upon application of capsaicin in presence of SB366791.

**Pre-application of SB366791 on effect of capsaicin (Caps)**											
**Parameter**	**Control**			**20 μM SB366791**			**10 μM Caps + 20 μM SB366791**			**Statistical Significance Control vs. 20 μM SB366791**	**Statistical significance Control vs. 10 μM Caps**
	**Mean**	**±SD**	***n***	**Mean**	**±SD**	***n***	**Mean**	**±SD**	***n***		
Amplitude (pA)	−23.1	4.1	6	−21.8	2.6	6	−21	6.2	6	ns, *p* = 0.18	ns, *p* = 0.49
Half-width (ms)	1.9	0.7	6	2.0	0.6	6	2.3	1.2	6	ns, *p* = 0.53	ns, *p* = 0.44
Rise time (ms)	1.0	0.6	6	1.1	0.2	6	1.2	0.4	6	ns, *p* = 0.55	ns, *p* = 0.55
Frequency (Hz)	2.55	0.8	6	1.98	0.7	6	4.96	1.5	6	ns, *p* = 0.063	^**^p = 0.0016
I_holding_ (pA)	−58.89	65.6	6	−58.96	56.4	6	−78.54	62.0	6	ns, *p* = 0.99	ns, *p* = 0.34

### Capsaicin does not alter evoked EPSC parameters or paired-pulse ratio (PPR)

**Figure 5:**
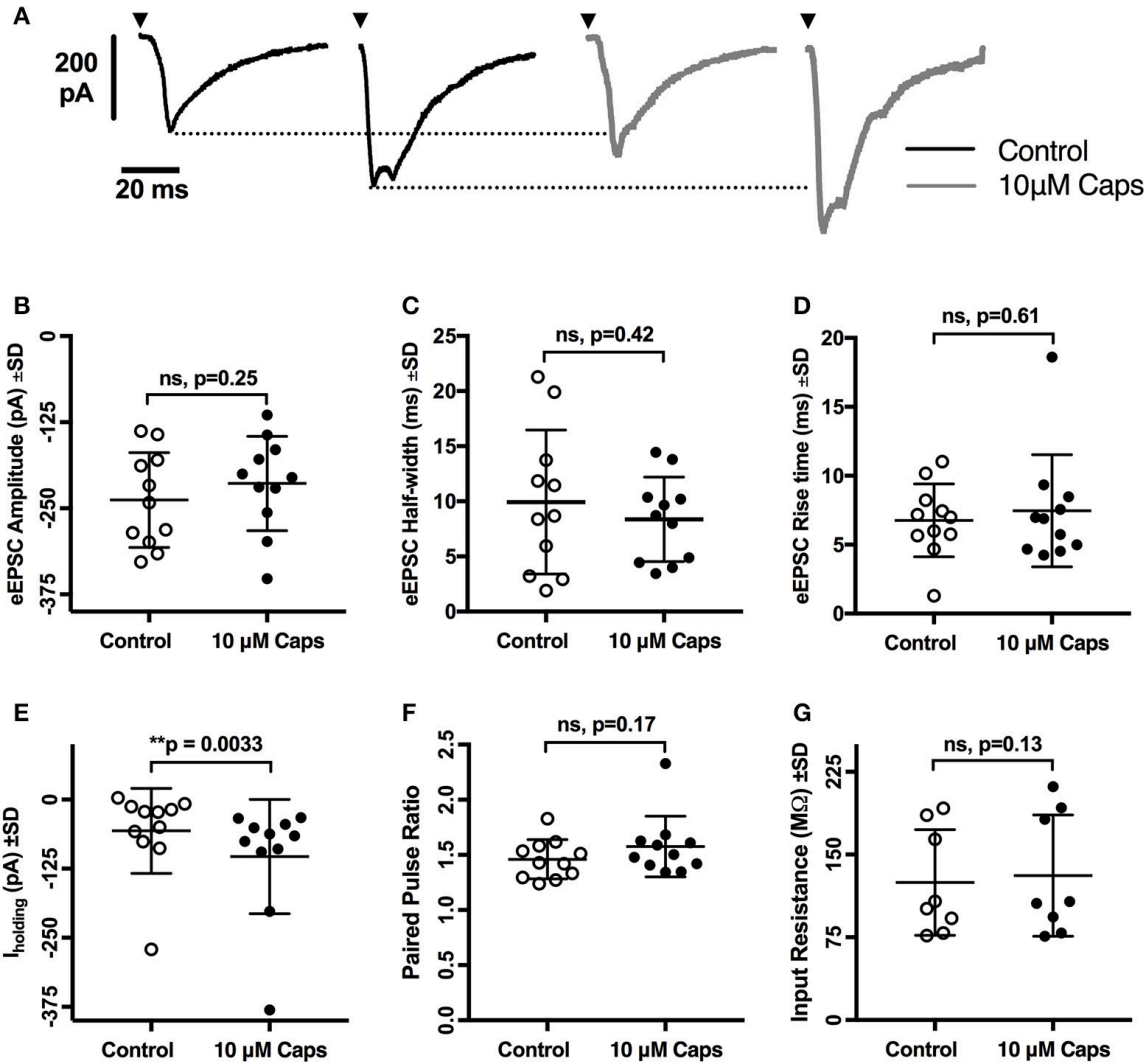
Evoked excitatory post-synaptic currents (eEPSCs) to rat hypoglossal motor neurons (HMNs) were unchanged after application of capsaicin (Caps). A pair of stimulus currents of 0.5–1.1 mA and 0.1 ms duration was applied to evoke a pair of eEPSCs. After recording baseline control responses, slices were superfused with capsaicin (10 μM) in aCSF for 9–11 min before recording the capsaicin-induced eEPSC responses. **(A)** Examples of averaged eEPSCs traces in control or in presence of 10 μM capsaicin from HMN voltage-clamped at −60 mV. Black triangles represent the time of stimulus current. **(B)** eEPSC amplitude showed no change during capsaicin treatment. Similarly, no significant change is observed in **(C)** half-width, **(D)** rise time, **(F)** paired pulse ratio (EPSC_2_/EPSC_1_), and **(G)** input resistance. However, there was a significant shift in the baseline holding current **(E)**. 20 μM strychnine HCl was present during recordings. Data represented as mean ± S.D. for *n* = 8 cells for input resistance and *n* = 11 cells for all other parameters. Significance is shown as ^**^p < 0.01. Paired two-tailed *t*-test was used to determine statistical significance.

As capsaicin increased both sEPSCs and mEPSC frequency, capsaicin may act via a pre-synaptic mechanism that is not mediated via classic TRPV1 activation. To further differentiate between modulation of activity-dependent and -independent transmitter release, we analyzed the effects of capsaicin on evoked excitatory post-synaptic currents (eEPSCs). Two stimuli in the reticular formation lateral to the hypoglossal motor nucleus evoked a pair of short-latency multi-synapse excitatory currents (average first eEPSC amplitude = −237.9 ± 68.8 pA, *n* = 11) separated by 150 ms. As shown in Figure 5, the first eEPSC before and after application of capsaicin showed no change in amplitude (Figure 5B, Table 5), half-width (Figure 5C) and rise time (Figure 5D), suggesting that activity-dependent release is not altered by capsaicin. This interpretation was strengthened by a lack of effect of capsaicin on the PPR of the first and second eEPSCs (Figure 5F). As for our observations of sEPSCs and mEPSCs, there was a negative shift in I_holding_ while recording eEPSCs, but we found no associated change in HMN input resistance (Figure 5G).

**Table 5:** Evoked EPSC parameters of HMNs upon application of capsaicin.

**Effect of Capsaicin on evoked EPSCs**							
**Parameter**	**Control**			**10 μM Caps**			**Statistical Significance**
	**Mean**	**±SD**	***n***	**Mean**	**±SD**	***n***	
Amplitude (pA)	−237.9	68.8	11	−213.9	68.3	11	ns, *p* = 0.25
Half-width (ms)	9.9	6.5	11	8.3	3.8	11	ns, *p* = 0.42
Rise time (ms)	6.7	2.6	11	7.4	4.0	11	ns, *p* = 0.61
Paired Pulse Ratio	1.46	0.1	11	1.576	0.2	11	ns, *p* = 0.17
I_holding_ (pA)	−56.63	76.9	11	−103	103.4	11	^**^p = 0.0033
Input Resistance (MΩ)	124.7	47.9	08	130.9	55.0	08	ns, *p* = 0.13

### Capsaicin does not affect repetitive action potential (AP) firing of HMNs

**Figure 6:**
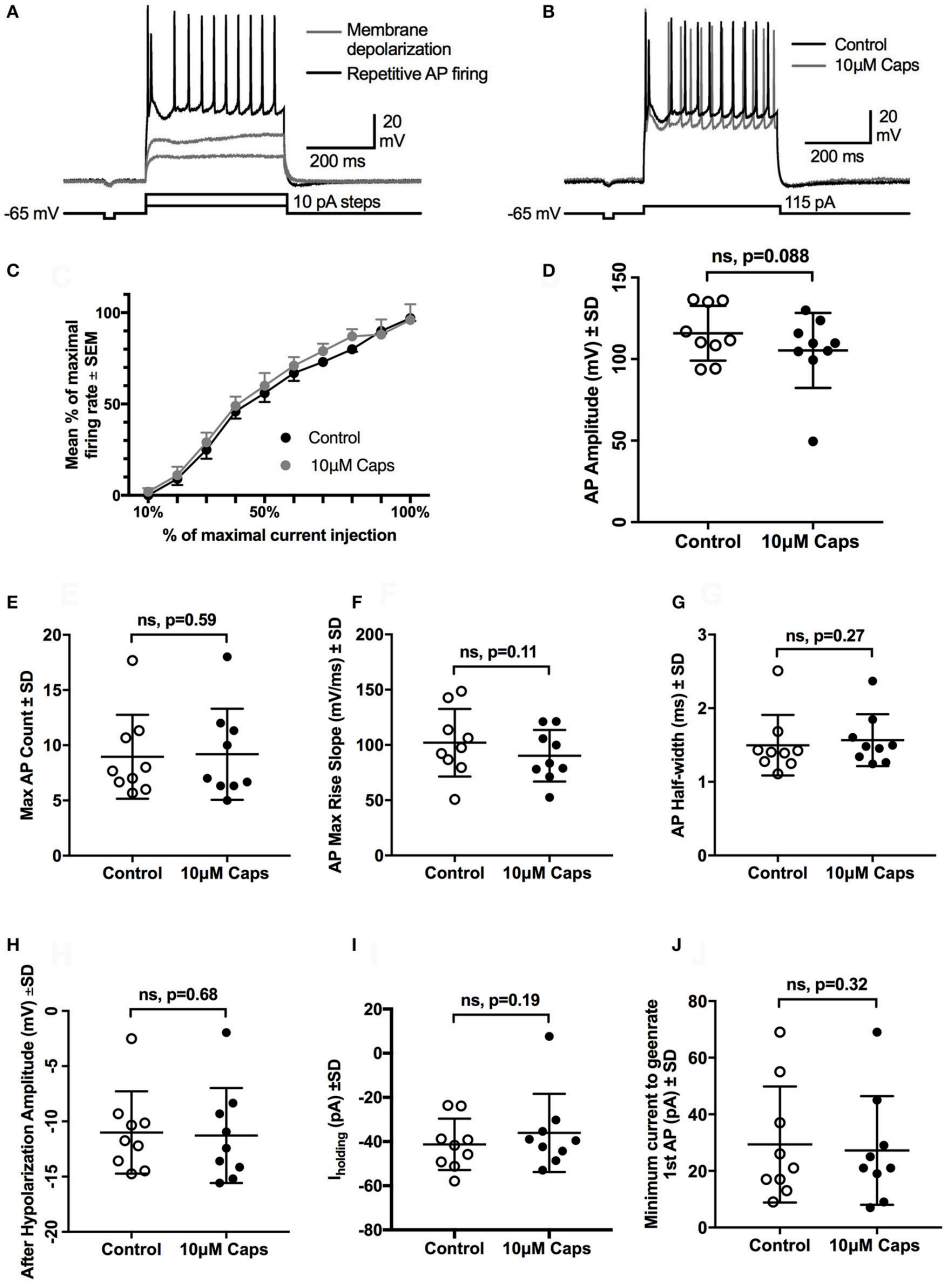
Repetitive action potential firing by HMNs is unchanged after capsaicin (Caps) application. **(A)** Depolarizing current steps caused steady-state membrane depolarization in the sub-threshold range (gray trace) and repetitive action potential firing in the supra-threshold range (black trace). **(B)** Capsaicin (10 μM) had no effect on the number of action potentials generated at the maximum amount of current injected. **(C)** Quantification of mean firing rate at the maximum current injected showed no change during capsaicin. All other action potential parameters, including amplitude **(D)**, rise slope **(F)**, half-width **(G)**, and after-hyperpolarization amplitude **(H)** remained unchanged. Action potential count **(E)** and minimum current required to generate first AP **(J)** also remained unchanged during capsaicin. Holding current (Ihold) was not significantly altered by capsaicin **(I)**. Data represented as mean ± S.E.M **(C)** or S.D. **(D–J)** for *n* = 9 cells, each from separate brain slice. Paired two-tailed *t*-test was used to determine statistical significance.

The effects of capsaicin on sEPSCs and mEPSCs strongly suggests it acts by increasing the release probability of pre-synaptic neuron terminals, with little postsynaptic effect on HMN excitability, although capsaicin consistently evoked an inward current in all conditions tested. To directly measure the effect of capsaicin on post-synaptic HMN excitability, we evoked repetitive action potential (AP) firing from HMNs current clamped at −65 mV, using a series of progressively increasing depolarizing current steps (Figure 6A, Table 6). Capsaicin did not change AP firing frequency in HMNs (*n* = 9, Figures 6B,C), nor did capsaicin change the number of APs evoked by maximum current injected in individual cells (Figure 6E). Analysis of shape parameters of APs under control and capsaicin conditions showed no significant change in AP amplitude (Figure 6D), rise slope (Figure 6F), half-width (Figure 6G) and after-hyperpolarization amplitude (Figure 6H). Capsaicin also had no effect on the minimum amount of current required to generate the first AP under both conditions tested (Figure 6J). However, it was interesting to note that under the recording conditions used for APs (holding potential, −65 mV and potassium methyl sulfate-based internal solution), there was no significant shift in I_holding_ (Figure 6I).

**Table 6:** Action Potential parameters of HMNs upon application of capsaicin.

**Effect of Capsaicin (Caps) on repetitive action potential firing**							
**Parameter**	**Control**			**10 μM Caps**			**Statistical significance**
	**Mean**	**±SD**	***n***	**Mean**	**±SD**	***n***	
AP Amplitude (mV)	115.8	16.8	9	105.3	23.0	9	ns, *p* = 0.088
AP Half-width (ms)	1.4	0.4	9	1.5	0.3	9	ns, *p* = 0.27
AP Max Rise slope (ms)	102.0	30.5	9	90.2	23.3	9	ns, *p* = 0.11
After Hypolarization Amplitude (mV)	−11.01	3.7	9	−11.27	4.3	9	ns, *p* = 0.68
I_holding_ (pA)	−41.25	11.6	9	−36.08	17.7	9	ns, *p* = 0.19
Max AP count	8.963	3.8	9	9.185	4.1	9	ns, *p* = 0.59
Min Current required to generate 1st AP (pA)	29.33	20.48	9	27.22	19.22	9	ns, *p* = 0.32

### Capsaicin significantly reduces the amplitude of spontaneous IPSCs (sIPSCs)

**Figure 7:**
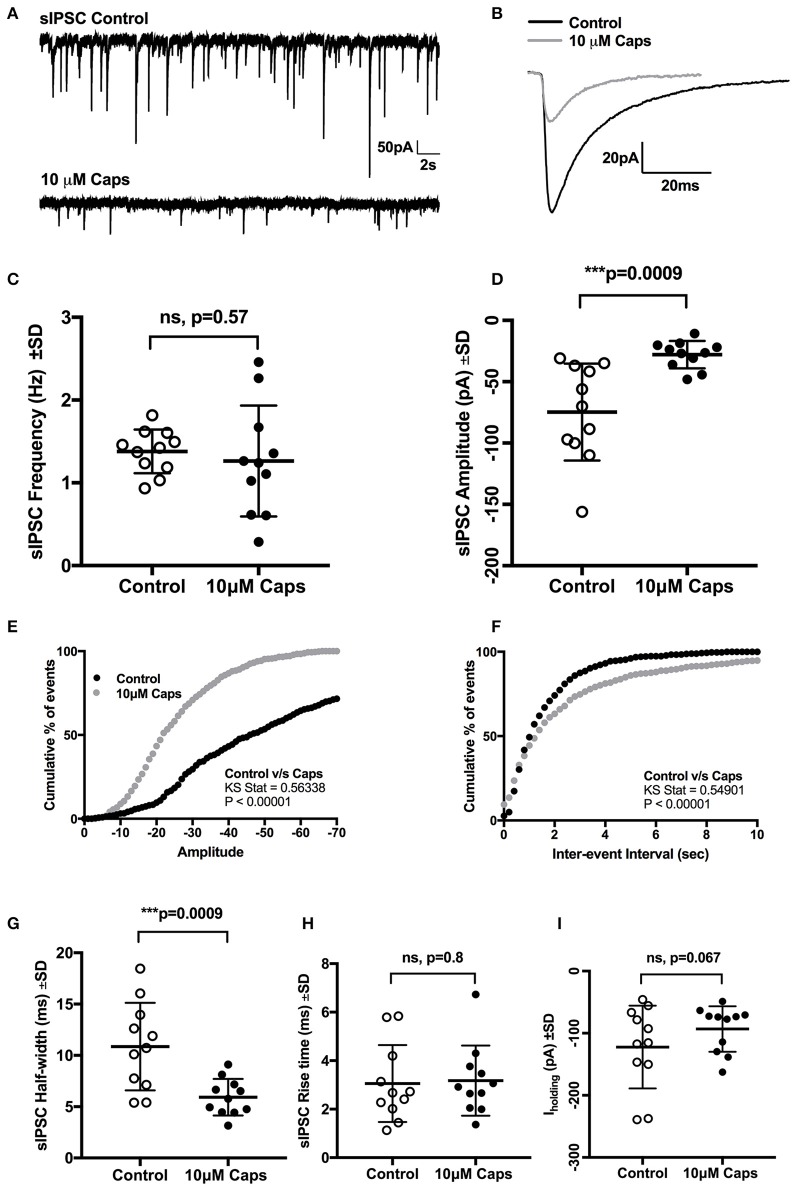
Capsaicin (Caps) reduces inhibitory synaptic transmission to rat HMNs. After recording baseline control activity, slices were superfused with capsaicin (10 μM) in aCSF for 9–11 min before recording the capsaicin induced spontaneous inhibitory post-synaptic current (sIPSC) activity. **(A)** Representative sIPSCs recorded in (control) and in 10 μM capsaicin from HMNs voltage-clamped at −60 mV. **(B)** Representative averaged traces of spontaneous IPSCs before (control) and during capsaicin. **(C)** Mean spontaneous IPSC frequency during control and capsaicin conditions, showing no significant change in frequency with capsaicin. **(D)** Mean spontaneous IPSC amplitude during control and capsaicin, showing a significant decrease in amplitude with capsaicin. Averaged cumulative frequency distribution of sIPSC amplitude **(E)** and inter-event interval **(F)**. Kolmogorov-Smirnov test to amplitude and inter-event interval showed a significant difference between control and capsaicin conditions. Mean sIPSC half-width **(G)** also showed a significant reduction after capsaicin application. Rise time **(H)** and baseline I_holding_ **(I)** remain unchanged after capsaicin application. All recordings were made with NBQX (10 μM), APV (50 μM) and bicuculline (5 μM). Data represented as mean ± S.D. for *n* = 11 cells, each from separate brain slice. Significance is shown as ^***^p < 0.001. Paired two-tailed *t*-test was used to determine statistical significance.

Our observation that sEPSC and mEPSC frequency was consistently increased by capsaicin raised the question of whether this effect was specific to glutamatergic presynaptic terminals or whether glycinergic synaptic terminals were also modulated. To investigate this, HMNs were voltage-clamped at a membrane potential of −60 mV and were recorded in aCSF containing a cocktail of NBQX (10 μM), APV (50 μM) and bicuculline (5 μM) to isolate glycinergic sIPSCs. During capsaicin application (9–11 min, 10 μM), there was a significant decrease in sIPSC amplitude (Figures 7A,B, Table 7), without any effect on sIPSC frequency (Figure 7C). Decreased sIPSC amplitude is illustrated by representative continuous traces in Figure 7A. Figure 7B shows averaged sIPSCs from control and capsaicin conditions, illustrating the decreased amplitude with capsaicin (−62% of control, *p* = 0.0009, *n* = 11; Figure 7D). Mean sIPSC half-width was also significantly reduced (−45% of control, *p* = 0.0009, *n* = 11; Figure 7G, Table 7). Figure 7E shows the average distribution of sIPSC amplitude, indicating a shift to smaller amplitude events in the presence of capsaicin. The average distribution of sIPSC inter-event intervals (Figure 7F) showed no significant shift between control and capsaicin conditions consistent with an unchanged frequency. Other sIPSC parameters, such as rise time (Figure 7H) and baseline holding current (I_holding_) (Figure 7I), were unchanged with capsaicin application, although I_holding_, was shifted toward more positive levels, opposite to the change observed during sEPSC recordings.

**Table 7:** Spontaneous IPSC parameters of HMNs upon application of capsaicin.

**Effect of Capsaicin (Caps) on spontaneous IPSCs**							
**Parameter**	**Control**			**10 μM Caps**			**Statistical significance**
	**Mean**	**±SD**	***n***	**Mean**	**±SD**	***N***	
Amplitude (pA)	−74.7	39.4	11	−27.9	11.1	11	^***^p = 0.0009
Half-width (ms)	10.8	4.2	11	5.9	1.7	11	^***^p = 0.0009
Rise time (ms)	3.0	1.5	11	3.1	1.4	11	ns, *p* = 0.8
Frequency (Hz)	1.37	0.2	11	1.26	0.6	11	ns, *p* = 0.57
I_holding_ (pA)	−122.3	66.6	11	−93.18	36.5	11	ns, *p* = 0.067

## Discussion

The key finding of the present study is that capsaicin enhanced the frequency of spontaneous and miniature excitatory synaptic currents to rat HMNs *in vitro*, but did not alter evoked excitatory synaptic currents, consistent with pre-synaptic enhancement of action potential-independent release probability. The effect of capsaicin on excitatory synaptic current frequency were not blocked by either of two different TRPV1 antagonists, suggesting that this effect is via a mechanism that does not require TRPV1 receptor activation. We also show that capsaicin significantly decreased spontaneous glycinergic inhibitory synaptic current amplitude, but not frequency, consistent with a postsynaptic effect on glycine receptor activity.

In the quantal theory of neurotransmitter release, a change in amplitude of miniature synaptic currents is considered to reflect an alteration in post-synaptic neurotransmitter receptor activity, while a change in the frequency of miniature synaptic currents indicates a change in action potential-independent pre-synaptic release probability (Redman, 1990; Stevens, 1993; Isaacson and Walmsley, 1995). Because capsaicin increased mEPSC frequency without altering mESPC amplitude, we interpret this as an enhancement of action potential-independent release probability. Capsaicin also increased sEPSC frequency, raising the possibility that action potential-dependent glutamate release might also be enhanced. However, we did not find any significant effects of capsaicin on evoked EPSCs, suggesting that capsaicin did not modulate action potential-dependent glutamate release. As spontaneous synaptic currents are a mixture of action potential-independent miniature currents, and action potential-dependent currents, we conclude that increased sEPSC frequency is due to increased mEPSC frequency.

We consistently observed an inward current in response to capsaicin during mEPSC, sEPSC and evoked EPSC recordings. However, this inward current was absent in our recordings of spontaneous IPSCs, when glutamate receptor antagonists were present, and was not associated with any significant change in input resistance. The most conservative mechanism for this inward current is an increase in tonic activation of glutamate receptors, presumably secondary to increased mEPSC frequency. This raises the question of where this input comes from. HMNs receive strong glutamatergic inputs from several regions, including respiratory central drive from Dbx1 positive interneurons in the reticular formation (Koizumi et al., 2008; Revill et al., 2015). It is likely that these last order premotor interneurons also integrate inputs from vagal afferent-driven NTS neurons (Bailey et al., 2002; Bailey and Fregosi, 2006; Beaumont et al., 2017), as well as TRPV1 positive trigeminal somatosensory neurons (Cavanaugh et al., 2011), as *in vivo* studies show that respiratory rhythm drives and modulates both whisking and sniffing behaviors (Moore et al., 2013, 2014). Recently, capsaicin, acting via TRPV1 receptors, has been shown to cause potent inhibition of the respiratory central pattern generator in the rhythmically active brainstem-spinal cord *in vitro* preparation and in plethysmographic *in vivo* recordings, but had no effect in a rhythmically active brainstem slice (Ren et al., 2017). Interestingly, respiratory apnoea was often accompanied by increased tonic motor activity (Ren et al., 2017), which is consistent with our interpretation, and with a study by Lee and colleagues, where increased tonic hypoglossal discharge was reported (Lee et al., 2003) at higher concentrations of capsaicin in anaesthetized rats.

However, other structures may provide capsaicin-sensitive afferent inputs to HMNs. TRPV1-positive vagal nerve afferents arborize extensively in structures adjacent to the hypoglossal motor nucleus, including the dorsal motor nucleus of the vagus, and the nucleus of the solitary tract (Kalia and Sullivan, 1982; Hermes et al., 2016). Synaptic transmission to neurons in these structures is modulated by capsaicin, increasing excitatory spontaneous release (Shoudai et al., 2010) and reducing miniature inhibitory post-synaptic currents (Xu and Smith, 2015). It is possible that these modulated vagal afferents may contribute to the enhancement of glutamatergic transmission by capsaicin in HMNs. As capsaicin took around 9–11 min to produce effects in our study, it is also possible that the effect seen in HMNs may be mediated by spread of a secondary messenger, such as endocannabinoids or endovanilloids.

The vanilloid capsaicin is one of the five major capsaicinoids present in chili peppers (Barbero et al., 2014). The effects of capsaicin are usually attributed to activation of TRPV1 channels (Caterina et al., 1997; Messeguer et al., 2006), and are blocked by specific antagonists of TRPV1. In our study, we used capsazepine, an antagonist of TRPV1 channels (Marinelli et al., 2002, 2003), to see if its pre-application can block the effect of capsaicin on sEPSCs. Although capsazepine is a competitive antagonist of TRPV1 receptors (Docherty et al., 1997), capsazepine can act on several other ion channels and receptors, including voltage-activated calcium channels (Docherty et al., 1997), nicotinic acetylcholine receptors (Liu and Simon, 1997), and hyperpolarisation-activated cyclic nucleotide-gated channels (Gunthorpe et al., 2004). Capsazepine also shows species-related variable effects on calcium influx (Savidge et al., 2002) and hence may be a less potent TRPV1 antagonist in mouse and rat (Correll et al., 2004). Although capsazepine did block the effect of capsaicin on sEPSC amplitude, it did not block the effects on sEPSC frequency and I_holding_. These results suggest capsaicin increases action potential-independent glutamatergic transmission by a mechanism other than TRPV1 activation. Our interpretation is further supported by reports that capsaicin elicited increases in sEPSC frequency in both wild type and TRPV1 knock-out mice (Benninger et al., 2008), indicating that modulation of excitatory synaptic transmission by capsaicin is not always mediated by TRPV1 channels.

Although capsaicin is a highly potent agonist for the TRPV1 receptor, some of the actions of capsaicin may not be mediated by TRPV1 receptors. Capsaicin is reported to directly inhibit voltage-gated sodium (Balla et al., 2001; Liu et al., 2001; Lundbaek et al., 2005; Cao et al., 2007; Wang et al., 2007) as well as calcium (Köfalvi et al., 2006) channels. As we saw presynaptic effects of capsaicin in the presence of TTX, we can rule out direct modulation of voltage-gated sodium channels, while the absence of any change in evoked EPSC amplitude or PPR strongly suggests that capsaicin does not directly modulate presynaptic Ca^2+^ channels. Other potential off target effects of capsaicin also include inhibition of transient (type A) and sustained (delayed rectifier type) voltage gated potassium channels in mouse trigeminal ganglion neurons, and these effects of capsaicin persisted in TRPV1 knockout mice (Yang et al., 2014). Both type A and delayed rectifier potassium currents contribute to regulation of rat HMN firing (Viana et al., 1993), which was unaltered by capsaicin. However, it remains possible that capsaicin reduces presynaptic potassium channel activity through a TRPV1-independent action, and this increases glutamatergic release probability. Furthermore, in human embryonic kidney (HEK) cells expressing TRPV1, capsaicin inhibited protein synthesis and depolymerized microtubules (Han et al., 2007). Additional effects of capsaicin at high doses are direct inhibition of the mitochondrial respiratory chain (Shimomura et al., 1989), significant decrease in mitochondrial membrane potential (Dedov et al., 2001), and actions as mitochondrial inhibitors to activate apoptosis and/or necrosis (Dedov et al., 2001).

It was interesting to note that capsazepine alone had significant effects; it decreased sEPSC amplitude, suggesting that there might be tonic activation of TRPV1 channels, or that there are non-specific actions of capsazepine (Yamamura et al., 2004). Another interesting finding was that sEPSC half-width was significantly increased by capsaicin in the presence of capsazepine, an effect that was not observed following capsaicin application alone. To address this issue, we tested the effect of another TRPV1 antagonist, SB366791. As for capsazepine, SB366791 did not block the effect of capsaicin on sEPSC frequency; however, SB366791 did block the change in sEPSC amplitude and in I_holding._ No changes were observed on any of the sEPSC shape parameters after SB366791. It seems likely that any tonic effect on sEPSC amplitude is largely by postsynaptic TRPV1 modulation.

We confirmed this by testing the effect of capsaicin on eEPSCs. Capsaicin did not change any eEPSC shape parameters or the eEPSC PPR. Only I_holding_ was altered significantly, consistent with prior results on spontaneous and miniature EPSCs. We note that there is a scattered population of TRPV1 positive interneurons in the reticular formation (Mezey et al., 2000), raising the possibility that our electrical simulation protocol failed to activate these TRPV1-sensitive neurons.

The negative shift in baseline holding current (I_holding_) elicited by capsaicin might alter HMN intrinsic excitability and action potential firing rate. We thus tested whether capsaicin altered repetitive action potential (AP) firing by HMNs. Capsaicin did not alter repetitive firing in HMNs nor were there any changes in action potential shape parameters. These results indicate that capsaicin does not strongly modulate motor neuron intrinsic excitability.

Finally, we tested whether capsaicin may also modulate inhibitory transmission to HMNs, which receive strong glycinergic inputs from local interneurons. By contrast to its effects on sEPSCs, capsaicin selectively and significantly decreased sIPSCs amplitude and half-width without altering sIPSC frequency. It was also interesting to note that I_holding_ was not significant altered, and shifted toward more positive value, opposite to the I_holding_ shift observed while recording sEPSCs. This result suggests that capsaicin significantly reduces glycinergic inhibitory synaptic transmission to rat HMNs by an entirely post-synaptic mechanism of action.

Both capsazepine and SB366791 blocked the increase in sEPSC amplitude elicited by capsaicin, indicating that this effect of capsaicin is mediated by TRPV1 activation. Noxious peripheral stimulation causes rapid translocation of GluR1-containing AMPA receptors to the cell membrane (Galan et al., 2004; Larsson and Broman, 2008) and phosphorylation of GluR1 subunits (Fang et al., 2003a,b), suggesting possible mechanisms driving increases in sEPSC amplitude. However, increased sEPSC and mEPSC frequency after capsaicin were not blocked by prior application of TRPV1 antagonists, ruling out a role for TRPV1 in this effect.

In conclusion, we have shown that capsaicin, a classical TRPV1 agonist, significantly modulates synaptic transmission to HMNs in brain slice preparations without affecting the intrinsic excitability of HMNs. We also show that these effects of capsaicin are largely not mediated by classic TRPV1 activation. This study adds to our present understanding of mechanism of action of capsaicin in central neurons and the possibility of non-TRPV1-mediated effects of capsaicin.

## Author contributions

MB and PT designed experiments, analyzed results, and wrote and edited the manuscript. PT carried out experiments.

### Conflict of interest statement

The authors declare that the research was conducted in the absence of any commercial or financial relationships that could be construed as a potential conflict of interest.
